# Assessing the Evolution of Gene Expression Using Microarray Data

**DOI:** 10.4137/ebo.s628

**Published:** 2008-04-24

**Authors:** Owen Z. Woody, Andrew C. Doxey, Brendan J. McConkey

**Affiliations:** Department of Biology, University of Waterloo, Waterloo, Ontario Canada

**Keywords:** functional evolution, gene expression, microarrays, gene duplication

## Abstract

Classical studies of the evolution of gene function have predominantly focused on mutations within protein coding regions. With the advent of microarrays, however, it has become possible to evaluate the transcriptional activity of a gene as an additional characteristic of function. Recent studies have revealed an equally important role for gene regulation in the retention and evolution of duplicate genes. Here we review approaches to assessing the evolution of gene expression using microarray data, and discuss potential influences on expression divergence. Currently, there are no established standards on how best to identify and quantify instances of expression divergence. There have also been few efforts to date that incorporate suspected influences into mathematical models of expression divergence. Such developments will be crucial to a comprehensive understanding of the role gene duplications and expression evolution play in the emergence of complex traits and functional diversity. An integrative approach to gene family evolution, including both orthologous and paralogous genes, has the potential to bring strong predictive power both to the functional annotation of extant proteins and to the inference of functional characteristics of ancestral gene family members.

## Introduction

Since the advent of molecular phylogenetics roughly fifty years ago, techniques in molecular evolution have relied predominantly on sequence information in order to model the evolutionary history of genes. Phylogenetic trees are based on an alignment of DNA or protein sequences, from which the architecture of gene duplication as well as evolutionary distances (branch lengths) between genes can be inferred. However, while a phylogenetic tree models the history of gene duplication events, it does not by itself reflect the evolution of gene function. As more is learned about the regulatory and structural complexity that dictates gene/protein function, it is becoming increasingly clear that additional non-sequence information is important to consider for a more complete understanding of the evolution of function in gene families.

One important characteristic of function that is poorly represented by sequence data alone is the transcriptional behavior of a gene. A gene’s transcriptional (or expression) profile may contain critical characteristics of function, including when and where a gene is expressed, and the conditions under which gene expression is induced. These regulatory properties may be crucial in explaining the key functional differences between related genes whose functions cannot be distinguished from sequence alone (e.g. Daugaard, Rohde and Jaattela, 2007), and thus more adequately reflect the functional diversity achieved within gene families (see for example Wang, Chong and Wang, 2006). Although attempts have been made to predict expression patterns of genes using sequence information (e.g. Beer and Tavazoie, 2004), most previous efforts have typically been limited to simple expression patterns controlled by known cis-elements. Due to the complexity and diversity of factors influencing gene regulation (e.g. Akitaya et al. 2003), automatic, sequence-based prediction of a gene’s temporal and spatial regulation remains a premature goal.

Microarray technology allows for a direct, quantitative measurement of a cell’s transcriptional response to a given stimulus, and is currently the most useful experimental source of large-scale gene expression data (Ranz and Machado, 2006). Microarrays are emerging as the mainstream technology for measuring gene expression due to their comprehensive coverage and relative cost-effectiveness (Ranz and Machado, 2006). Data sets spanning a wide selection of organisms under various treatment and developmental conditions are publicly available, providing a ready source of data for many aspects of gene transcription behavior.

The integration of genomic and transcriptomic data is providing an increasingly detailed picture of molecular evolution by incorporating regulatory behavior into models of the evolution of gene expression and function. Here we review recent work in the field, including methodologies for investigating gene expression evolution, studies concerning the influence of various factors on expression divergence, and promising recent developments that address expression divergence from a gene family perspective. The implications for future studies of the evolution of gene expression are also discussed.

## Evolutionary Changes in Gene Expression

Gene expression, measured by detecting the presence and quantity of transcribed mRNA, is often an initial step in a cell’s response to a given condition or stimulus. When the expression response of a cell is measured with respect to numerous conditions, the transcript abundance for each gene in these experiments is combined to produce an expression profile. The expression profile therefore summarizes the transcriptional state of a gene in response to the measured experimental stimuli, but is relative to the specific subset of conditions being measured and compared. The characteristics of gene regulation typically captured in expression profiles include dosage (abundance), location (tissue/locus), time/event of activation (treatment/control status), and duration of expression in the case of repeated measures. The subset of microarray data captured within a profile might reflect multiple biological characteristics for a given gene, as genes may have multiple modular functions each under the control of separable regulatory programs (McClintock, Kheirbek and Prince, 2002). Expression divergence may be defined as evolutionary changes to these regulatory programs, as inferred through comparison of the profiles of homologous genes.

**Figure 1:**
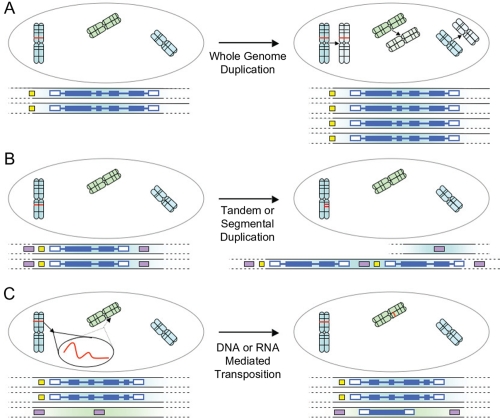
Common modes of gene duplication. The chromosome complement of the cell with the genomic location of the gene (red) and an expanded schematic of the region containing the duplicated gene (blue) are shown, as well as *cis*-regulatory sequences (yellow) and arbitrary sequence markers (purple). **A**) Whole genome duplication, resulting in a doubling of gene copy number. All *cis*-regulatory elements are preserved. **B**) Tandem or segmental duplication, respectively producing local or large-scale duplications/deletions. In the example shown, unequal crossing over results in duplication on one strand and a deletion on the other. *Cis*-regulatory elements may also be copied along with duplicated gene(s). **C**) Transposition, mediated by RNA (retrotransposons) or DNA (DNA transposons), resulting in sequence being copied to a new genomic location. In the example, transposition is mediated by an mRNA transcript of the original gene, and associated *cis*-regulatory sequences and introns are not copied.

Evolutionary changes in gene expression may occur through numerous mechanisms. In the simplest case, gene expression may be altered by mutations in cis-regulatory regions (Smith et al. 2006). Mutations affecting genes elsewhere in the regulatory network may also result in expression divergence, for instance through changes in the regulation of a transcription factor or coding-sequence mutations that can affect interactions with other proteins or DNA (Wang et al. 2007; Xing et al. 2007). Furthermore, some duplication events may be considered mechanisms for expression divergence (Cannon et al. 2004). Duplications whereby only partial upstream segments are copied may lose *cis*-elements and thus cause shifts in gene expression (Haberer et al. 2004). Similar situations might occur in RNA-mediated duplications where the upstream regions may be lost altogether. These modes of duplication are in contrast to whole-genome duplication events in which upstream regions are kept intact. In short, changes in expression can be partially predicted from changes in upstream sequence, but are also affected by other mechanisms which are frequently difficult to predict (Wang et al. 2007). Typical mechanisms of gene duplication are shown in Figure 1.

The change in a gene’s expression behavior over time can be measured in two somewhat contrasting ways. Evolution of gene expression can be measured directly by observing state changes over the course of generations, or historical states and state changes can be inferred using extant gene expression profiles and an appropriate model of evolution. Equivalently, gene expression evolution can either be observed in real time or past events can be inferred from existing data. Three common methodological approaches for measuring gene expression evolution/divergence are discussed below.

### Mutation accumulation

Mutation accumulation studies are a commonly used approach to study the evolution of gene expression in real time. These studies of expression evolution attempt to accelerate the rate of change to a level that can be observed in the lab by providing an organism with nutrient rich, threat-reduced media which minimizes selective pressure. Mutation accumulation studies can be used to compare wild-type organisms to counterparts which have been raised in a nutrient-rich and threat-free medium. Under this relaxed selection, any mutations that are not severely deleterious are more likely to become fixed in sufficiently small populations, leading to observable expression divergence and differences in expression patterns between strains.

### Ortholog divergence

Inferring historical changes in gene expression, however, may be more relevant as these changes may be of significant evolutionary importance. Here, studies of expression evolution take advantage of existing orthologous genes as points of comparison. As orthologous genes are separated by a speciation event, they date back to a common structure and function in the pre-speciation ancestor. By examining orthologs, one can investigate how expression has diverged from this state in the two organisms post-speciation. This may reflect the contribution of neutral drift (Khaitovich et al. 2004; Whitehead and Crawford, 2006a; Whitehead and Crawford, 2006b), and how gene expression has evolved in response to similar or slightly different selective pressures on the two species.

### Paralog divergence

Lastly, gene expression evolution may also be investigated in the context of paralogs—duplicate genes residing within the same host genome. Paralogs present an opportunity to study how expression has diverged following a gene duplication event, where two copies of a gene may result in some degree of functional redundancy. In classical studies of gene duplication (e.g. Ohno, 1970), the fates of these duplicated genes were interpreted by the gain and loss of function. This approach has given rise to terms such as nonfunctionalization, subfunctionalization, and neofunctionalization, which describe the gain and/or loss of protein function subsequent to a duplication event. These terms can be applied equally well to expression evolution; specifically, nonfunctionalization corresponds to a single gene losing its expression behavior in one or more circumstances, subfunctionalization denotes different and complementary losses of expression behaviors in paralogs, and neofunctionalization corresponds to a gene acquiring a novel regulatory characteristic (Tirosh and Barkai, 2007).

**Figure 2:**
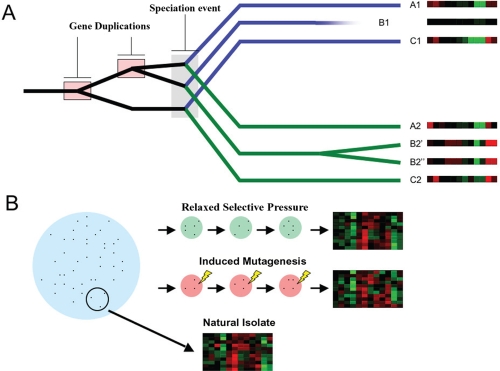
Methods for evaluating expression evolution and divergence. **A**) Comparison of expression profiles of extant orthologs and paralogs. A simple sequence-based gene tree is shown for two extant species, including single gene duplication, gene deletion/inactivation, and speciation events. Present day paralogs are the result of single gene duplications occurring prior to (e.g. A1/C1) or subsequent to (e.g. B2′/B2″) speciation. Orthologs (e.g. A1/A2) are separated due to speciation. Sample expression profiles for individual genes under different conditions are shown as heat maps (right), and may be used to assess expression divergence and conservation. **B**) Induced expression evolution. Expression evolution can be accelerated to an observable rate in organisms with short generation times. Changes in expression relative to natural isolates can be produced in small populations exposed to several generations of either minimal selective pressure or active mutagenesis, and these changes assessed by comparison of expression profiles for multiple genes.

The above three means for studying expression evolution, summarized in Figure 2, correspond to different paradigms of selective pressure on genomes encountered over the natural course of evolution. Changes in functional profiles occur in parallel to changes in sequence. Sequence divergence is the standard means for measuring evolutionary distance separating homologous genes. Standard sequence-motivated phylogenetic trees may be used to determine the historical time-frame for speciation/duplication events, allowing any observed expression evolution to be dated and interpreted in context with other major evolutionary events.

## Quantification and Comparison of Expression Profiles

Given the gene expression profiles of two or more homologs or a given gene studied across mutation accumulation products, a next question is how to distinguish expression divergence from conservation. Differences in expression profiles can be measured in terms of the presence or absence of expression across tissues or in response to stimuli, relative shifts in expression in response to stimuli, or the abundance of transcribed mRNA in each measured condition. Unfortunately, there are no *a priori* means to identify which aspects of gene expression response (timing, magnitude, or location) are most crucial. As such, metrics of divergence may have different or conflicting interpretations. For many purposes, simple presence/absence data can be sufficient—for example, a gene expression profile could be defined as the set of tissues and/or responses in which the gene is expressed above a given threshold. In this framework, the expression profiles of two genes can be compared by contrasting their presence/absence across several conditions and/or tissue types.

A more detailed description of expression behavior can be derived by quantifying transcript abundance. Gene expression signals (i.e. microarray spot intensities) can be used either to measure absolute abundance by condition or to contrast differences in expression across two or more conditions. Ideally, absolute expression could be used to provide unbiased and broadly comparable counts of produced transcripts. However, absolute signal intensity has been shown to be subject to a wide variety of biases (lab, technician, scanner, and probe sequence effects, to name a few) which limit the interpretability of raw expression intensity (Irizarry et al. 2006). Ratios of signal intensities across conditions are generally more broadly comparable, though they may obscure expression shifts with widespread effects (for example, a ratio of 1 does not distinguish between high expression in both tissues and minimal expression in both tissues).

The relative strengths of these metrics are still largely undetermined. A study by Yang et al. (2005) contrasted the advantages of abundance-based metrics with those based on presence/absence data. Their data set consisted of different human and mouse tissues under a normal, no-stimulus condition. While both metrics showed merit, each had circumstances under which its interpretation could be misleading.

A further possible issue with microarray data arises when assessing closely related (and thus highly similar) genes. There is a risk of cross-hybridization for similar sequences, particularly in older arrays or those with sub-optimal probe designs. This could introduce an artifactual correlation between highly similar genes that could be falsely interpreted as expression correlation. Commercial microarrays are periodically updated with new probes and probe-to-gene associations (Dai et al. 2005; Roche et al. 2004) that reflect more recent and accurate builds of genome sequences, which should presumably reduce this potential effect.

In addition to methods for handling individual microarray data sets, a reliable metric is also needed to quantify the extent of expression divergence between pairs of profiles. Gu et al. (2002), for example, used pairwise correlation coefficients between expression profiles to represent profile similarity. Alternatively, other studies have used a weighted difference of expression levels across the two profiles by either taking differences in abundances or ‘response’ sizes (Casneuf et al. 2006; Ha, Li and Chen, 2007). More advanced metrics employ ANOVA frameworks to attempt to partition expression profile variability into multiple components (Nuzhdin et al. 2004; Duarte et al. 2006).

## Evolution of Expression in Orthologs and Paralogs

In the absence of selective pressure, gene expression profiles can randomly drift over time (Wagner, 2000). Some of the first studies using microarray expression data to examine regulatory evolution sought to address whether neutral drift is a primary means of divergence, or, if not, what parameters might cause gene expression to diverge beyond a basal rate (Wagner, 2000; Gu et al. 2002; Makova and Li, 2003).

In one of the initial studies to examine the rate of change in paralogs, Wagner (2000) evaluated expression divergence by correlating profile correlations with sequence distances. The study employed 20 selected paralog pairs with a high degree of sequence and expression profile similarity that dated to a particular genome duplication in *S. cerevisiae*, and found a weak but non-significant correlation between expression divergence and sequence divergence in these closely related pairs.

This work was extended by Gu et al. (2002), who expanded the dataset of Wagner (2000) to include all closest paralogs in the genome for which microarray data were available. The authors further evaluated a pair of alternative measures of sequence similarity tracking either synonymous or non-synonymous substitutions. Consistent with Wagner (2000), most duplicates showed little correlation between sequence and expression divergence in the long-term. However, they noted that a statistically significant correlation could be obtained by focusing exclusively on paralogs with very small sequence dissimilarities. The similarity of two expression profiles was evaluated by correlating the expression levels of a pair of genes across various conditions (cell cycle stages, budding, and responses to experimental stimuli totaling 208 response data points per gene). These expression profile correlations were themselves correlated with both synonymous or non-synonymous sequence divergence between the pair of genes. This procedure revealed a weak but significant correlation between sequence and expression divergence when only recent duplications (those with sequence dissimilarities below a K_A_ value of 0.3) were examined. A likely explanation for this weak but significant correlation is that the data likely contain a mixture of genes with correlated and non-correlated expression profiles. Thus, further classification of the data may be required before consistent patterns may be observed in the evolution of gene expression.

Nuhzdin et al. (2004) examined expression profile divergence in orthologs from two closely related species of Drosophila, *D. melanogaster* and *D. simulans*. Profiles were built from normal-state expression sampled from whole-fly mRNA. An ANOVA model was used to capture the specific component of variance corresponding to expression divergence across species, and the magnitude of this variability was compared with sequence divergence across the two species. They observed a moderate correlation between expression profile divergence and non-synonymous sequence divergence. Sequence divergence had no detectable relationship with expression divergence when only synonymous substitutions were considered, suggesting that proteins under higher sequence constraint also exhibited higher constraint on expression divergence.

The studies examined thus far indicate a relationship between sequence and expression evolution in both orthologs and paralogs. However, the weak correlations that have been detected suggest that other factors may be influencing expression evolution on a gene-by-gene basis. Subsequent studies described below examine the sequence/expression relationship in more detail and have identified other factors affecting rates of expression divergence.

If additional factors can be accounted for, it could clarify the relationship between sequence divergence and expression divergence and explain much of the variability found in correlational studies (e.g. Guan, Dunham and Troyanskaya, 2007). These factors may also identify features that can be incorporated into predictive models of expression divergence and functional annotation, as well as models for reconstructing ancestral expression profiles (Lemos et al. 2005a). The following sections highlight some of these factors (summarized in Table 1).

## Factors Influencing Expression Divergence

### Modes of gene duplication

Gene duplications can occur via numerous mechanisms (Fig. 1) including unequal crossover, whole genome duplications, and retrotranspositions (Zhang, 2003). Depending on the mode of duplication, a newly copied gene may or may not retain its cis-regulatory information. Thus, it follows that rates of expression divergence are also likely to be similarly affected.

In a correlation-based study of factors influencing expression divergence in *A. thaliana*, Casneuf et al. (2006) split paralogs into two groups: one composed primarily of duplicated genes produced in a whole genome duplication (WGD) event, and the other composed of tandem repeat duplicates. The expression profiles between paralogs were evaluated using Spearman correlation coefficients based on microarray data spanning a wide variety of experimental conditions. It was generally found that tandem duplicates had divergent expression profiles and that typically only one gene amongst a tandem series exhibited widespread expression across a variety of tissues, whereas most others in the set were expressed only in specific conditions. Paralogs produced by whole genome duplication events, on the other hand, showed stronger profile correlations and similar expression breadth (i.e. both paralogs were expressed in several tissues under several circumstances, and were not in general specialized to a specific niche purpose).

Subsequent explorations of the effects of duplication mode were performed on paralogs within the polygalacturonase gene family of *A. thaliana*. Kim et al. (2006) used PCR transcript abundance data from various *A. thaliana* tissues to derive their expression profiles. Despite evidence demonstrating that mode of duplication bears an influence on post-duplication divergence, the study found no evidence for a relationship between sequence and expression divergence when examining all possible sets of paralogous pairs in their specific gene family.

In another study (Haberer et al. 2004), *A. thaliana* duplicates were grouped based on their mode of duplication and expression levels were measured using massively parallel signature sequencing (Brenner et al. 2000) in a variety of tissues. The study found no evidence for the sequence/expression correlation in either segmental duplicates or tandem duplicates. They did not attempt to restrict the focus of their study to closely related duplicates, but their results do not suggest that the relationship would be any clearer for subsections of the data.

In a study of WGD in *S. cerevisiae,* Tirosh and Barkai (2007) found that the mode of duplication seemed to have a strong influence on duplicate retention. Specifically, duplicates produced in WGD events were more likely to demonstrate asymmetric divergence (with respect to the *C. albicans* ortholog) than smaller scale duplications. These findings are in contrast to those of Casneuf et al. (2006), who found that WGD events produced copies whose expression profiles were more correlated than those produced by smaller-scale duplications. This discrepancy may be due to differences in genome properties between plants and fungi. However, the comparisons themselves may not be directly analogous, as Tirosh and Barkai measured divergence with respect to a distant ancestral ortholog, whereas Casneuf gauged divergence using differences between paralogs themselves, a method that they acknowledge is poorly suited for comparing present day expression profiles to ancestral ones. In any case, this discrepancy highlights the difficulty in uncovering universally applicable rules for expression divergence, and motivates the inclusion of as much supplementary genomic data as possible.

Guan, Dunham and Troyanskaya (2007) also demonstrated that the relationship between sequence divergence and expression divergence differs depending on the mode of duplication. Using extensive information regarding the biological functions, expression patterns, genetic interactions and essentiality of duplicates in *S. cerevisiae*, fundamental differences were found between paralogs originating in WGD and paralogs from smaller-scale duplications (SSD). WGD paralogs shared more interaction partners, were less essential, showed more divergent expression, and were more frequently synthetically lethal when pairwise eliminated compared to SSD paralogs. Furthermore, relationships between sequence divergence and either functional similarity or expression divergence were present only in SSD paralogs. These distinctions between WGD and SSD were demonstrated to be independent of sequence divergence.

### Upstream sequences and TATA boxes

Recent studies have indicated that presence of the TATA box cis-regulatory element may be a strong predictor of mutability in expression patterns. Tirosh et al. (2006) examined expression divergence between orthologs in *S. cerevisiae* along with four other closely related and two distantly related species of yeast. Their measure of expression divergence was based on transcript abundances across a wide variety of stimuli. Differences were calculated between expression estimates for species pairs under each condition, and these differences were then summed to form an index of species-wise differences for each gene. By breaking genes into groups based on the presence of a TATA box, Tirosh et al. (2006) were able to detect convincing differences in expression divergence, with increased divergence being more common in the TATA-group. This effect was also present within individual functional categories of genes, and functional groups showing the most divergence also contained the greatest proportion of TATA-mediated genes.

Landry et al. (2007) also reported evidence for the effect of TATA-mediated expression divergence in *S. cerevisiae*. Using a mutation accumulation approach, the study found that two regulatory factors explained a large portion of observed divergence. They concluded that presence of a TATA box is a strong predictor of a propensity to diverge. The other influential factor was the number of trans-regulatory influences on a given gene’s expression—the greater the number of trans-regulatory interactions, the greater the potential for divergence. Why this relationship between TATA-presence and expression divergence exists is a bit of a mystery. Tirosh et al. (2006) speculate that it may be a means of flagging genes whose expression in a given tissue/scenario is free to experience neutral drift. It is possible the TATA box may itself directly affect the propensity for expression divergence, though the mechanism for this is not understood.

### Chromosomal position

Denver et al. (2005) examined expression datasets constructed from several independent lines of *C. elegans*, some of which were natural isolates and some of which were mutation accumulation lines. By comparing expression variability in the natural isolates versus the mutational lines, Denver et al. determined that while mutation accumulation lines accrued mutations equally across the length of the chromosome, variability in expression across natural isolate lines was mostly in genes located on autosomal arm regions and not in the core. While this may be evidence for an influence of chromosomal location on expression divergence, the authors point out that there is also a tendency for important, widely conserved genes to be located near the autosomal core, offering a viable alternative explanation.

### Gene function

Different genes are under different magnitudes and types of functional constraint and are therefore likely to diverge at different rates. Similarly, it would be expected that gene function may also significantly influence rates of expression divergence.

Makova and Li (2003) examined expression divergence in human duplicates, using tissue presence/absence to construct expression profiles. The Makova and Li dataset used human tissues under presumably normal conditions, where expression levels being compared were abundances with no reference to a control state. They found similar results to Gu (2002) when examining a correlation between expression divergence and protein sequence divergence. It was found that those gene families at the extremes of rapid divergence and tight conservation often corresponded to functional categories in which considerably high or low divergence rates would be expected from a biological perspective. For example, immune response was rapid to diverge, and transcription factors and enzymes tended to display conservation of expression patterns. Some functional families showed up in both categories, however.

Casneuf (2006) also noticed an apparent effect of function on rates of expression divergence. Two exceptions of particular interest were genes involved in “cell death” and “development”, as genes in these groups demonstrated no clear relationship between sequence and expression divergence.

### Tissue localization/breadth

A gene’s tissue expression pattern itself may have an effect on the susceptibility of expression to change, as constraints (e.g. developmental constraints) on gene expression may vary between tissues. In a recent study, Gu and Su (2007) elaborated on this concept, termed the “tissue-driven hypothesis”. They defined two metrics, *E*_ti_ (tissue expression distance between human and mouse) and *D*_ti_ (tissue protein sequence distance between human and mouse), and found that *E*_ti_ and *D*_ti_ were highly correlated when they concerned the same tissues. For genes with similar tissue-breadth (number of tissues in which the gene is expressed), higher rates of expression divergence were found in tissues with relaxed developmental constraints than those under a tightly-controlled developmental program such as brain tissues.

Tissue-breadth is therefore also a potential factor in expression divergence, as broadly versus narrowly expressed genes may vary in degree of constraint. Yang et al. (2005) noted that the propensity for orthologous genes to diverge in expression was dependent on tissue. Divergence between human and mouse was measured, using evidence of transcription in a tissue as a criterion for function. Using a novel index of expression conservation, the Expression Conservation Index (ECI), Yang et al. (2005) measured the proportion of tissues shared in common by two genes as a fraction of their pooled tissue breadth (i.e. the intersection over the union). The study demonstrated that expression profiles tend to evolve more rapidly for genes which are expressed in only a limited number of tissues. This is consistent with Gu and Su’s (2007) tissue-driven hypothesis, as tissue-specific genes may be under more tissue-specific/developmental constraints than broadly expressed genes.

### Age of gene family

Freilich et al. (2005) conducted a study to evaluate the relative contributions of gene family age and function on expression divergence. The study involved a paralog analysis conducted on mouse, and presence calls were used to describe the expression profile of genes across several tissue types. While a relationship was detected between function and rates of expression divergence, the effect could be explained by the age of the protein family in question—specifically, ancient proteins tend to be expressed in many or all tissue types, while recent proteins specific to metazoa or mammalia tend to have a more refined and tissue-specific breadth of expression.

### Protein length and interactions

In a paper investigating factors that influence mRNA expression evolution and variability within a species, Lemos et al. (2005a) observed expression levels in *D. pseudoobscura* and several strains of *D. melanogaster*. Divergence was measured by a normalized difference in expression levels, whereas polymorphism was measured as within-species expression level variability in D. melanogaster.

Both protein size and the number of protein interaction partners were found to correlate negatively with gene expression divergence and variability. These relationships remained intact even when other possible confounding factors were eliminated. These findings make intuitive sense, as small protein products with few interactions are presumably less likely to produce detrimental effects following a change in expression pattern, and therefore such changes in pattern are more likely to become fixed.

## Limitations of Pairwise Studies

There is accumulating evidence that analyses of closest pairs may not be sufficient to determine the complete story behind expression evolution. Freilich et al. (2006) have noted that while paralogs tend to adopt narrow expression profiles and specialize towards a select few expression locations and stimuli, the general trend for the entire gene family is to retain a wide breadth. This may have some implications for results based on pairwise data. For example, it may be possible to distinguish between neofunctionalization and subfunctionalization by studying what functions have been represented elsewhere in the gene family. If a gene re-acquires expression in a tissue in which ancestral genes were expressed, this form of neofunctionalization may be more adequately described as a reversion than an innovation. In the absence of gene family data, one must ultimately guess whether a difference in expression across homologs is a gain of function in one or a loss of function in the other.

While it may be true that closest relatives will be most likely to share similar expression characteristics, the presence of similar expression characteristics in distantly related genes is a potentially interesting factor that is not addressed in most expression studies. Doxey et al. (2007) examined expression profiles from an entire gene family, and noted the presence of several common expression patterns in subsets of extant genes despite variable mutational distance separating their duplication and divergence. In pairwise studies, the relative value of each difference in expression profile is equivalent, whereas a family-wide perspective reveals trends in conservation and loss that can help explain differences encountered in pairwise studies.

Although studies of paralogous and orthologous gene pairs are well-suited to reveal factors important in influencing expression evolution, they run the risk of overlooking phenomena and trends experienced by the gene family as a whole. A comprehensive analysis of expression evolution within a gene family, however, can address some of these issues, such as conservation of function between more distant homologs.

### Frameworks for ancestral character estimation

In light of these limitations, a logical next step may be to expand the focus of studies from pairwise duplicates to gene families within an organism or across related species (i.e. spanning orthologs and paralogs). While this increase in scope may help resolve trends in expression evolution, it comes at the cost of additional complexity in determining relatedness and evaluating plausible ancestral characters. Two alternative methods for inferring ancestral expression profiles of paralogs exist at present: estimation from extant gene expression profiles, or making use of orthologous genes in a species which predates/lacks the duplication event—i.e. species where the ‘ancestral’ gene still exists.

Tirosh and Barkai (2007) conducted a study focused on a set of *S. cerevisiae*-specific paralogous pairs for which a single *C. albicans* ortholog could be identified, i.e. an ortholog that had not undergone further duplication in *C. albicans*. The objective was to look for similarities in expression profiles between the *S. cerevisiae* paralogs and the *C. albicans* gene. Asymmetric divergence would appear as one paralog matching the *C. albicans* ortholog to a significantly greater degree than the other. Several paralogs in *S. cerevisiae* had expression patterns similar to each other and their corresponding *C. albicans* ortholog, and these pairs tended to also have relatively high expression levels, which the authors suggested might correspond to selection for dosage. These genes were examined and found to have minimal effects in gene-knockdown studies done elsewhere (Giaever et al. 2002). Intriguingly, they also found a group of paralogs composed of one gene matching the *C. albicans* ortholog and one with a divergent expression profile. These cases were termed to be instances of ‘regulatory neofunctionalization’—the adoption of a new expression profile to become selectively significant.

Huminiecki and Wolfe (2004) also examined whether expression profiles of orthologs differed if there was a lineage-specific duplication following speciation. They found that, in general, presence of a paralog in either lineage resulted in reduced agreement between both paralogs and the corresponding unduplicated ortholog. In a majority of cases, these paralogs adopted more restrictive profiles and often lost functions compared to their common ancestor.

Another example of the benefits of a more comprehensive approach to analyzing gene expression evolution is presented in the work of Gu et al. (2005). Their objective was to examine gene families as a unit, with their ancestry and relatedness modeled by a phylogenetic tree. The functions of each extant gene were modeled in the family using information from a database of *S. cerevisiae* regulatory interactions. The ancestral genes (internal nodes in the tree) were then annotated with an interaction complement estimated by parsimony reconstruction. This contrasted post-duplication genes against their inferred parental profile, which in turn allowed detection of phenomena such as asymmetric divergence and neofunctionalization. These phenomena are difficult to identify in studies that focus exclusively on gene pairs.

**Figure 3:**
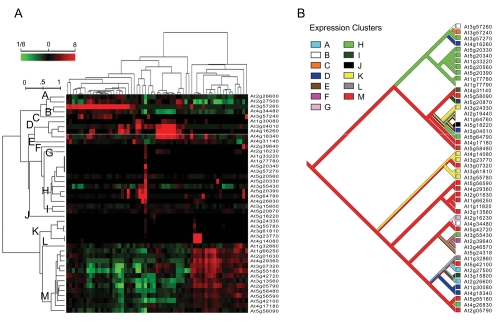
Inference of ancestral expression changes by mapping expression data onto a gene tree. In this example, microarray data (Schmid et al. 2005) is used to map expression changes in the *A. thaliana* beta-1,3-glucanase gene family (adapted from Doxey et al. 2007). **A**) Classification of genes using expression profiles. Individual experiments (tissue profiles) are shown vertically and genes are shown horizontally, with expression quantified relative to the average for each given gene. Genes with similar profiles are assigned into expression clusters, which can be used as characters in phylogenetic reconstruction. **B**) Parsimony was used to reconstruct ancestral expression profiles from the defined expression classes. In this example, a notable feature is the sub-tree showing a high degree of conservation of class H (green), suggesting an evolutionary innovation on this branch. Here, class H is associated with fl owering, functionally distinct from the inferred ancestral class M.

In another study utilizing phylogenetic methods, Doxey et al. (2007) investigated expression in the *A. thaliana* β-1,3-glucanase (BG) gene family. Using expression data from a series of tissue development and stress response experiments (measured as differences relative to a pooled sample and an unperturbed control plant, respectively), the authors sought to uncover similarities in expression profiles amongst existing plant BG genes and to use these similarities to infer the function of extant and ancestral genes. To detect similarities, genes were clustered according to their expression profiles. These cluster assignments were then used as characters for reconstruction on the BG phylogenetic tree using a parsimony-based procedure, which demonstrated conservation of expression within this family and additionally inferred ancestral expression patterns. Some ancestral expression divergence events corresponded to major events in the evolution of BG function, such as the origin of common activity within a subfamily. An illustration of the methodology used for functional annotation the gene family-level is shown in Figure 3.

Integrative studies allow the natural application of expression profiles to a gene family, as depicted through a phylogenetic tree. By tracking the properties of genes in this tree, it should be possible to make informed predictions about the expression profiles of extant but unannotated family members, as well as predictions about expression profiles of ancestral genes.

## Discussion

### Consequences for analysis of gene evolution and functional annotation

The fact that expression divergence between homologs does not necessarily correspond to sequence divergence has profound implications for functional annotation. Specifically, while sequence homology serves as a strong predictor of molecular function, the regulatory information concerning how, when, and where this function is applied (i.e. biological process) cannot be based on sequence alone. Important regulatory information can be incorporated from analysis of expression behavior within the gene family, along with identification of potential confounding factors. This more holistic approach is consistent with Eisen’s (1998) concept of phylogenomics— the functional annotation of genes from a phylogenetic perspective. By incorporating information across entire gene families, pooling paralogs and orthologs, it should be possible to obtain a detailed description of function and its evolutionary history.

The call to adopt a phylogenomic perspective has been made previously (Eisen, 1998; Eisen and Wu, 2002; Balandraud et al. 2005; Brown and Sjolander, 2006). The augmentation of phylogenomics with gene expression profiles would result in more precise descriptions of function and evolutionary history. This call for improvement is similar to the suggestion of including structural modeling as a factor in determining whether genes are sufficiently homologous to allow a transfer of functional annotation (Sjolander, 2004). Although these approaches require some additional computational cost, recent work suggests that such an augmentation is possible. For example, Huertas-Cepas et al. (2007) recently described what was termed the complete human “phylome”, which is composed of phylogenetic trees mapping every gene family in the human genome. If the phylome were combined with large-scale expression data, genome-wide phylogenetic reconstruction of expression profiles should be possible.

Khaitovich et al. (2006) recently proposed that the Human HapMap project, an effort to identify sequence polymorphisms through extensive sequencing of the human population, could benefit considerably from the collection of expression assays in parallel to sequence. This transcriptome project would also address some of the concerns discussed here—for example, it would provide a more natural estimate of the background expression divergence expected under a relatively uniform amount of selective pressure.

### A common definition for expression divergence and profiling

At present, studies of expression evolution tend to make use of their own measurement schemes, and the relative merits of these metrics of conservation/divergence have not been fully assessed. This causes some difficulty in extending given results beyond their immediate context. A systematic comparison of the relative stringencies and sensitivities of existing metrics would be invaluable as a foundation for standardization of future studies. Although it may often be the case that no one metric will be ideal, having knowledge of the relative behavior of available measures will be useful for designing future experiments.

It would be valuable to evaluate the relative importance of factors influencing expression evolution in a given context. A relative evaluation through a dimension reduction technique (e.g. Drummond et al. 2006) could be used to uncover relationships between the variables presented above, and could reduce or simplify the factors to monitor in any future analyses. At present, it is also relatively difficult to incorporate time series data into a gene’s expression profile. Typically, each time point or group of similar time points is considered as an individual condition or tissue. While this approach simplifies analysis, it may overlook interesting sequential expression patterns unless the results are examined in detail (Kim et al. 2006). Recent work by Sahoo et al. (2007) suggests a means of recording expression responses in a time series by specifically noting the time points corresponding to activation and inactivation. This simplification still captures a significant portion of the information in a time series, and allows time to be summarized as a pattern amenable to reconstruction on a phylogenetic tree.

Alternatively, it may be interesting to extend current work on reconstructing ancestral expression clusters through the use of biclustering, which attempts to find subsets of genes and conditions/tissues in which there is a pattern of coordinated expression (Prelic et al. 2006). This method has the advantage that individual genes can be assigned to multiple biclusters, hopefully providing a better reflection of each of the biological processes in which they are involved.

### Applications of ancestral expression

Barring the availability of an orthologous, non-duplicated gene to use as a proxy, the most common means of inferring ancestral expression profiles is to estimate them from profiles of extant genes. A number of models for expression evolution have been proposed for ancestral character estimation (Gu, 2004; Guo et al. 2007; Oakley et al. 2005), and some software has been made available (Rossnes, Eidhammer and Liberles, 2005). These methods can estimate the probability of various modes of duplication, and additionally should be capable of accurately inferring ancestral expression states.

There are many potential uses for ancestral expression profiles. Beyond the reconstruction of the ancestral function of the entire gene family, it may possible to annotate major innovations in the tree (Doxey et al. 2007; Sjolander, 2004)—i.e. particular expression-related evolutionary adaptations. Similarly, ancestral expression profiles may identify cases of functional reversion, addressing the relatively unexplored issue of whether there are expression behaviors that are periodically lost and reacquired. Additionally, the identification of a high degree of similarity between distant branches of tree could provide evidence for the existence of synfunctionalization, the horizontal transfer of functions from one homolog to a sibling (Gitelman, 2007).

## Conclusion

Studies of gene expression evolution have highlighted many key factors influencing the fate of function following changes in selective pressure. As our understanding of gene expression evolution improves, it should become possible to incorporate expression profiles as a means to infer protein function. Ancestral protein functions can be estimated using this approach, and efforts to annotate current genes/proteins will benefit from knowledge of the behavior of and factors influencing expression profiles. Ultimately, expression profiles should be equally integrated with structure and sequence to predict and assist in annotating protein function and evolution.

## Figures and Tables

**Table 1:** Literature sources discussing influences affecting expression evolution.

	Mutation accumulation lines	Ortholog studies	Paralog studies
**Relationship between sequence and expression divergence**		Nuzhdin et al. 2004	Gu et al. 2002
			Casneuf et al. 2006
			Makova, Li, 2003
			Wagner, 2000
			Guan et al. 2007
**TATA box presence**	Landry et al. 2007	Tirosh et al. 2006	
**Gene function**	Denver et al. 2005		Casneuf et al. 2006
			Makova, Li, 2003
**Tissues constraints**		Yang, Su and Li, 2005	Makova, Li, 2003
		Gu, Su, 2007	Haberer et al. 2004
**Chromosomal location**	Denver et al. 2005		
**Mode of duplication**			Kim et al. 2006
			Guan et al. 2007
			Casneuf et al. 2006
**Age of gene family**			Freilich et al. 2005
**Gene complexity**		Lemos et al. 2005a	
		Lemos et al. 2005b	
